# Impact of COVID-19 lockdown on A&E performances in an NHS Foundation Trust

**DOI:** 10.1136/postgradmedj-2020-138894

**Published:** 2020-09-10

**Authors:** Sushrut Oomman, Elizabeth Todd

**Affiliations:** Emergency Medicine, County Durham and Darlington NHS Foundation Trust, Durham, UK; Emergency Department, County Durham and Darlington NHS Foundation Trust, Durham, UK

## INTRODUCTION

County Durham and Darlington Foundation Trust (CDDFT) is a National Health Service (NHS) Trust and is one of the largest integrated care providers in England. It serves a population of around 650 000 people.^1^ The two major acute hospitals in this Trust are the University Hospital of North Durham (UHND) and Darlington Memorial Hospital (DMH). Both of those hospitals have type 1 Accident and Emergency (A&E) departments,^2 3^ which means that they are a consultant lead 24-hour service with full resuscitation facilities and designated accommodation for the reception of accident and emergency patients.

During the COVID-19 pandemic, strategies were put in place to deal with spread of the virus. In the UK, there was an attempt to flatten the curve so that the NHS would be able to deal with sicker patients and provide all the support needed. To enable this, lockdown was introduced with social distancing, stay at home message and advice to wash hands regularly in order to break the cycle of spread of the virus.

The NHS also restructured all the hospitals in order to prepare for the crisis. All the wards in UHND and DMH were sectioned into red and blue zones to aid with the patient flow. The A&E in both UHND and DMH too was split into red and blue areas. All patients were triaged according to their symptoms; red area was for patients with respiratory or COVID-19 symptoms, and blue area was for patients with non-respiratory symptoms. Doctors were redeployed from the community placements and other non-essential rotations to A&E and other essential wards. All the elective and non-emergency operations were halted during the lockdown. Outpatient departments were also closed during the lockdown period.

A&E performance in the UK is measured via the ‘golden 4-hour standard’ and the ‘clinical quality indicators’. The 4-hour standard aims to assess, treat, admit or discharge patients within 4 hours and have been in place in the UK since early 2000s.^3 4^ Clinical quality indicators have been in place since 2010 and include the ‘total time spent by patients in the A&E department before moving to a ward, transferring to another hospital or clinical unit, or being discharged’, the ‘time to initial assessment of the patient’, the ‘time to treatment of the patient’, the ‘rate of the number of people who left without being seen and assessed’ and the ‘unplanned patient reattendance rate’.^5^

## AIM

I looked at A&E performances both before and during lockdown implementation.^6^ CDDFT has been collecting data from A&E in both UHND and DMH on clinical quality indicators since 2011 and has also been monitoring the 4-hour performance on a monthly basis.

The CDDFT data was compared to the NHS performance summary which is compiled by NHS England.^7^

## STANDARDS

The 4-hour A&E waiting time target is a pledge set out in the Handbook to the NHS Constitution. The operational standard is that at least 95% of patients attending A&E should be admitted, transferred or discharged within 4 hours.^3^

Another standard used is the document produced by the Royal College of Emergency Medicine—‘Emergency department clinical quality indicators: a CEM guide to implementation’ (March 2011).^5^

## METHODOLOGY

A review was carried out on CDDFT database on A&E statistics and clinical quality indicators. This will help us compare CDDFT data to the NHS performance summary.

### CDDFT database

Table 1 shows the overall CDDFT performance in all the clinical quality indicators and how the trend has shifted from the prelockdown period to the lockdown period.^6^

**Table 1:** Clinical quality indicator performance for CDDFT

A&E clinical quality indicators							
Trust performance summary—acute monthly				February 2020	March 2020	April 2020	May 2020
County Durham & Darlington NHS Foundation Trust—ED activity	Total attendances			10 878	9336	6779	9037
	HQU09: A&E clinical quality—unplanned reattendance- rate			6.3%	6.0%	7.1%	6.7%
	HQU10: A&E clinical quality—total time in the A&E department	Admitted patients	Median	06:09	04:39	03:21	03:23
			95th percentile (standard=04:00)	**14:30**	11:42	05:44	05:28
			Single longest time recorded	23:17	19:55	12:28	11:42
		Non-admitted patients	Median	03:09	02:51	02:12	02:10
			95th percentile (standard=04:00)	08:07	06:51	03:59	03:58
			Single longest time recorded	19:55	22:19	13:26	12:59
		All patients	Median	03:40	03:18	02:34	02:32
			95th percentile (standard=04:00)	10:44	08:32	04:46	03:59
			Single longest time recorded	23:17	22:19	13:26	12:59
	HQU11: A&E clinical quality—left without being seen rate			5.1%	4.3%	**1.3%**	**1.5%**
	HQU12: A&E clinical quality—time to initial assessment, patients arriving by emergency ambulance		Median	00:08	00:06	00:04	00:04
			95th percentile (standard=00:15)	**01:14**	00:51	**00:23**	**00:25**
			Single longest time recorded	04:26	03:24	01:34	01:50
	HQU13: A&E clinical quality—time to treatment		Median (standard =01:00)	01:46	01:17	**00:30**	**00:37**
			95th percentile	05:20	04:23	01:49	02:08
			Single longest time recorded	09:46	06:57	05:50	08:43

The total A&E attendance in both UHND and DMH combined in February 2020 was 10 878. This decreased to 6779 in April 2020.^6^ This suggests that A&E attendance had decreased during this period possibly due to lockdown being one of the factors at play. In April 2019, the total attendance was 11 718, so in April 2020 the attendance had dropped by 42% in comparison to April 2019.

The 95th percentile for the total time spent by all patients in A&E was 10 hours and 44 min in February 2020, and this dropped to 4 hours and 46 min in April 2020. In April 2019, it was 8 hours and 22 min. Correlating with the decreased patient number in April 2020, the data suggests that this may be influenced by the effect of lockdown on A&E attendance leading to better patient flow and patients getting served in a timely manner. Also, other factors might be at play like the availability of hospital ward beds causing no exit blocks contributing to better patient flow.

### Time to initial assessment and treatment in CDDFT A&E

The standard for the ‘time to initial assessment’ in A&E is 15 min. According to hospital data, since 2011, it has never been met.

In February 2020, the 95th percentile for the ‘time to initial assessment’ in CDDFT was 1 hour and 14 min. This then decreased to 51 min in March. In April, the time to initial assessment went right down to 23 min,^6^ which again may be influenced by the reduced number of patients attending A&E as well as other factors such as no exit block, better patient flow and adequate staffing leading to a better doctor to patient ratio. Therefore, more patients were seen in a timely manner during the lockdown period. The 95th percentile for ‘time to initial assessment’ in April 2019 was 39 min, which is off the standard.

The standard for the ‘time to treatment’ is 1 hour, so that in 1 hour the patient should be assessed and have had the treatment started. In February 2020, the median value was 1 hour and 46 min and went down to 30 min in April 2020.^6^ In April 2019, the median value for ‘time to treatment’ was 1 hour and 16 min.

### Four-hour target performance in CDDFT A&E

In February 2020, 60.40% of the combined A&Es in CDDFT met the 4-hour target, and in March, this increased to 70.27%, and by April 2020, it had risen to 93.92%.^6^ In April 2019, it was 77.70%. This may be because more patients were seen and treated in a timely manner after the lockdown was implemented; the reasons for this could be multifactorial as stated above.

### A&E attendances in NHS England

Across NHS, a similar picture was seen. A&E attendances for the first time have fallen below 1 million in the month of April 2020.^7 8^ This may be because the patients probably felt that they should stay away from hospitals and keep it available for COVID-19 patients. On the other hand, A&E attendances before the lockdown period were steadily rising from 2010 when it was above 1.5 million until December 2019 to more than 2 million.

Statistics from NHS England show that in April 2020, 9.6% of patients attending A&E spent more than 4 hours from arrival to admission, transfer or discharge.^7 8^ The worst level on record was in December 2019 at around 30%.

## DISCUSSION AND CONCLUSION

The observed results suggest that the reduced patient number may have been influenced by the fear of catching COVID-19 in the hospital environment. People may have been reluctant to attend hospital.

The CDDFT performance against standards was very good during the lockdown. However, this is a temporary phase and is likely not a true reflection of performance as lower patient attendance and staff redeployment may have contributed to better patient flow and patients being served in a timely manner.

On reviewing the data, it seems that CDDFT is following a similar trend as other Trusts in NHS England. This effect can be reviewed again during the post-lokdown period.
